# The Association between Tensiomyography and Elastography Stiffness Measurements in Lower Limb Skeletal Muscles

**DOI:** 10.3390/s22031206

**Published:** 2022-02-05

**Authors:** Abdulrahman M. Alfuraih, Ahmed Alhowimel, Sara Alghanim, Yaaqoub Khayat, Abdulaziz Aljamaan, Hana I. Alsobayel

**Affiliations:** 1 Radiology and Medical Imaging Department, College of Applied Medical Sciences, Prince Sattam bin Abdulaziz University, Kharj 11942, Saudi Arabia; 2 Department of Health and Rehabilitation Sciences, College of Applied Medical Sciences, Prince Sattam bin Abdulaziz University, Kharj 11942, Saudi Arabia; a.alhowimel@psau.edu.sa; 3 Physiotrio Physical Therapy Clinic, Riyadh 13213, Saudi Arabia; 433203743@ksu.student.edu.sa (S.A.); ykhayyat@physiotrio.com (Y.K.); Aaljamaan@mhclinics.net (A.A.); 4 Department of Rehabilitation Sciences, College of Applied Medical Sciences, King Saud University, Riyadh 11451, Saudi Arabia; hsobayel@ksu.edu.sa; 5 Research Chair for Healthcare Innovation, Department of Rehabilitation Sciences, College of Applied Medical Sciences, King Saud University, Riyadh 11451, Saudi Arabia

**Keywords:** tensiomyography, elastography, muscles, reproducibility, shear wave

## Abstract

The objective was to test the measurements association between tensiomyography (TMG) and shear wave elastography (SWE) when evaluating the skeletal muscle stiffness of healthy subjects. The secondary objective was to evaluate the effect of superficial non-muscular tissues thickness on the measurements. A cross-sectional study was conducted with adults who are asymptomatic and had no previous history of musculoskeletal conditions. The vastus lateralis (VL) and biceps femoris (BF) muscle contraction was tested using TMG and SWE. The TMG parameters included time of contraction (Tc), sustain time (Ts), relaxation time (Tr), delay time (Td), and maximal displacement (Dm). The skin, subcutaneous fat, and fascia thicknesses were investigated using ultrasound imaging. A total of 25 participants were enrolled in the study. Six participants were females (24%). The mean age (SD) was 26.5 years (4.7). There was a statistically significant difference (*p* < 0.001) in SWE between VL (8.1 kPa) compared with the BF (10.8 kPa). As for Dm, which reflects stiffness in TMG, no difference was detected (*p* = 0.90), as both muscles had a maximum displacement of 3.7 mm. The correlation coefficients failed to detect any significant correlation (r ≤ 0.300, *p* ≥ 0.1) between SWE and TMG variables. There was no significant difference between male and female participants across all TMG and SWE variables (*p* > 0.10). Overall, there was no association between TMG parameters and SWE measurements, indicating that each technique might be evaluating a different biomechanical property of skeletal muscle.

## 1. Introduction

Skeletal muscles are complex structures with unique biomechanical properties. Stiffness in mechanical context is related to the resistance of a body against an applied force to change its length which involves deformable bodies possessing elastic energies [1,2]. Clinically, however, musculoskeletal stiffness can be described from the single muscle fiber to the entire system of cartilages, ligaments, muscles, and tendons combined [2]. Altered muscle stiffness can be associated with repetitive stress injuries and muscle strains [3,4].

There are several non-invasive techniques for assessing muscle stiffness. Shear wave elastography (SWE) is a relatively new technique based on ultrasound that can accurately and reliably estimate soft tissue stiffness [5]. It can detect altered muscle stiffness with increasing age [6], chronic neck pain [7], Duchenne muscular dystrophy [8], glucocorticoid treatment [9], and several other pathologies [10]. On the other hand, tensiomyography (TMG) is a device that can assess various contractile parameters using a displacement sensor activated by means of neuromuscular stimulation. A parameter, maximal radial muscle displacement (Dm), is particularly associated with stiffness [11,12]. TMG facilitates reliable and non-invasive assessment of skeletal muscles in athletes [11], for assessing low back pain [13], and for evaluating overall muscle function and fatigue [11,14]. A recent article reviewed the technique in-depth and discussed its role for several applications [12].

SWE involves placing a region of interest (ROI) measurement box over the investigated muscle followed by acoustic pulses that perturbate the local muscle fibers, which generates new a new form of waves, shear waves. Tracking the displacement such waves make can infer the stiffness information of the specific region within the muscle [5]. In contrast, TMG estimates the radial deformation of the whole muscle belly induced by an electrical stimulation which is tracked using a contact displacement sensor. This enables the plotting of a displacement-time curve to infer relevant parameters such as Dm. Hence, TMG is accepted as a method that evaluates the entire muscle contraction system including the tendon and myotendinous junction [11]. TMG is limited by several factors including the effect of the stimulation intensity and scarcity of studies reporting its external validity [12].

The relationship between TMG and SWE has not been thoroughly investigated beyond theory. We hypothesized that TMG demonstrates concurrent validity compared with SWE when assessing the biomechanical properties of skeletal muscle, and the measurements would not be significantly affected by the thickness of superficial non-muscular tissues. Hence the objective of this study was to compare the TMG outcomes against the SWE stiffness measurement on healthy subjects. A secondary objective was to evaluate the effect of skin thickness, subcutaneous fat thickness, and fascial layer thickness on the TMG and SWE measurements.

## 2. Materials and Methods

### 2.1. Study Design

The study was conducted as a cross-sectional study. All investigations were carried out in a sports rehabilitation center (PhysioTrio, Riyadh, Saudi Arabia) between October to November 2020. The study was approved by the review board at Prince Sattam bin Abdulaziz University. All participants provided informed consent prior to their inclusion in the study. Following convenience sampling, participants were invited from social media and from local sports societies as well as relatives and colleagues. They were eligible if they were asymptomatic, and 18–40 years of age. Participants were excluded if they had a history of muscle or joint injury history or were unable to provide consent. Each participant completed a survey to evaluate their physical activity. The demographics, height, and weight were also recorded. All participants underwent the ultrasound scan first prior to the TMG test. The investigations involved a knee extensor muscle (vastus lateralis (VL)) and a knee flexor muscle (biceps femoris (BF)). Both muscles were examined in a static and relaxed condition.

### 2.2. Elastography

**Figure 1:**
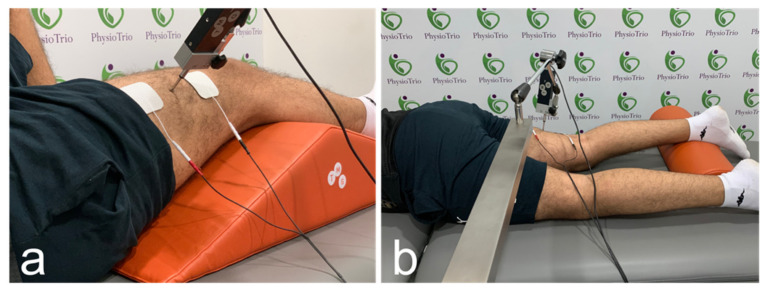
Acquisition locations for shear wave elastography and probe placements for tensiomyography during the assessments of the vastus lateralis (**a**) and biceps femoris (**b**).

Ultrasound imaging was acquired using a clinical scanner (Acuson Redwood; Siemens Healthcare, Mountain View, CA, USA) equipped with the 10L4 linear array transducer. To assess muscle stiffness, the machine uses virtual touch tissue imaging and quantification technology, where acoustic push pulses are sent to generate shear waves which are tracked using detection pulses to estimate the velocity of the shear waves. The shear waves velocities are later converted to Young’s modulus unit in kilo Pascals (kPa) to reflect tissues stiffness [5]. A circular region of interest with a diameter of 1 cm was selected in the belly of each muscle [15]. An operator with more than 5 years of experience in musculoskeletal elastography and 11 years of experience in ultrasonography recorded all SWE measurements. Participants rested for five minutes in a supine position prior to their SWE assessment. All measurements were performed in a resting state without any active muscle contraction or external stimulation. Measurement locations were marked with a dermatological pen to be reproduced in the TMG test. For the VL, the probe was placed at 30% of the length from the patella to the greater trochanter when the participant was in a supine position and the leg was rested on a wedge foam pad at 30° passive flexion (Figure 1a) [15,16]. For BF, the participant was in a prone position and the knee flexed by a 15° roll cushion; the probe was placed at 50% of the length between the ischial tuberosity and lateral condyle (Figure 1b) [17,18]. The transducer was placed on the skin with minimal pressure ensuring good contact and no external compression force. Due to the anisotropic anatomy of skeletal muscles, the transducer was oriented along the muscle fibers to yield reliable and valid results as recommended in previous studies [15,19]. Three repeated measurements were acquired for each muscle and the average was used for the analysis [17]. The measurements were recorded in kPa and checked for consistency in results to m/s.

### 2.3. Tensiomyography

The TMG S2 device (TMG Measurement System, TMG-BMC Ltd., Ljubljana, Slovenia) was used to record the tensiomyography measurements. The variables below were recorded to describe the functional involuntary contractile property of the examined muscle using TMG. Time of contraction (T_c_) represented the time between 10% and 90% of the maximum vertical muscle movement. Sustain time (T_s_) represented the time from 50% of the muscle contraction to 50% of its relaxation. Relaxation time (T_r_) represented the time required to reduce the muscle contraction, after an electrical stimulus, from 90% to 50% of the overall maximum muscle belly displacement. Delay time (T_d_) was the time required to reach 10% of the maximum muscle belly displacement. Maximal displacement (D_m_) represented the muscle’s overall displacement (shortening) by an involuntary stimulation. These times were expressed in milliseconds (ms) and the displacement in millimeters (mm).

TMG was performed according to the protocol by García-García et al. [11]. Two specialists in the TMG S2 device took the measurements; one monitored the strength and frequency of the administered stimulus, while the other was responsible for sensor installation and positioning. The positions are illustrated in Figure 1. Electrical stimulation was given with a monophasic pulse length of 1 ms and an initial current amplitude of 30 mA, which was gradually raised to 110 mA in 5-mA increments (maximal stimulator output). A ten-second rest time was used to separate consecutive stimuli [20].

### 2.4. Physical Activity

The International Physical Activity Questionnaire (IPAQ), short version, was used. It consists of seven items assessing different types of physical activity intensity and sitting time that people do as part of their daily lives. These items were considered to estimate total physical activity in MET-min/week and time spent sitting according to the guidelines from IPAQ (www.ipaq.ki.se, accessed on 2 September 2020) [21]. The final scores were ordinally divided into three levels of physical activity: low, medium, and high, according to the guidelines. The Arabic version of IPAQ was used which has high reliability and good concurrent and construct validity [22].

### 2.5. Statistical Methodology

Descriptive and inferential statistics were used to analyze the data using SPSS version 27 (Armonk, NY, USA: IBM Corp). The continuous data were tested for normality using the Shapiro–Wilk test and histogram evaluation. The association between SWE and TMG was tested using Pearson’s and Spearman’s correlation coefficients depending on data normality. These coefficients were also used to test the effect of superficial layers on the measurements. Intraclass correlation coefficients were used to verify the intra-operator reproducibility of SWE. Multiple linear regression was planned to understand the effect of superficial layers on the TMG measurements.

## 3. Results

**Figure 2:**
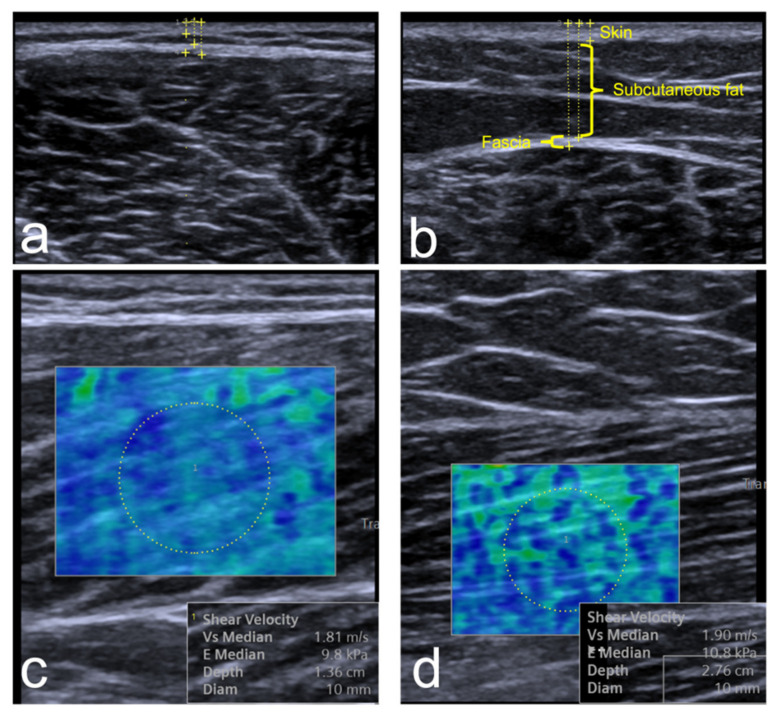
A sample of the elastography and ultrasound thickness measurements for the vastus lateralis (**a**,**c**) and biceps femoris (**b**,**d**). The thickness measurements show the calibers placement for skin, subcutaneous fat, and fascia. The SWE images show the hue color distribution of the stiffness and the Young’s modulus kPa results.

Overall, 76 volunteers agreed to take part in the study. Nine volunteers were excluded due to a history of MSK injuries and 42 did not arrive for their appointments. In total, 25 volunteers participated and were included in the study. Three participants refused to undergo TMG due to fear or feeling uncomfortable undergoing the test. Six participants were females (24%). The mean age (SD) was 26.5 years (4.7), ranging from 21 to 40 years. The mean BMI (SD) was 23.6 (3.2). Only three (12%) participants reported to be smokers. With regard to physical activity, five (20%) had low physical activity, eleven (44%) were moderate, and nine (36%) were high. The descriptive statistics for the ultrasound, elastography and TMG are listed in Table 1. Two variables (Ts and Tr) did not present normal distribution. Hence, they were reported as medians and evaluated using non-parametric statistics. The table shows that the mean stiffness of BF (10.8 kPa) was higher than VL (8.1 kPa) on SWE (*p* < 0.001). However, this difference in stiffness was not detected in the maximal displacement in TMG (*p* = 0.90). Sustain and relaxation times were significantly different between vastus lateralis and biceps femoris (*p* < 0.001 and *p* = 0.007, respectively). There was no significant difference between male and female participants across all TMG and SWE variables (*p* > 0.10). An example of the ultrasound and elastography is shown in Figure 2.

**Figure 3:**
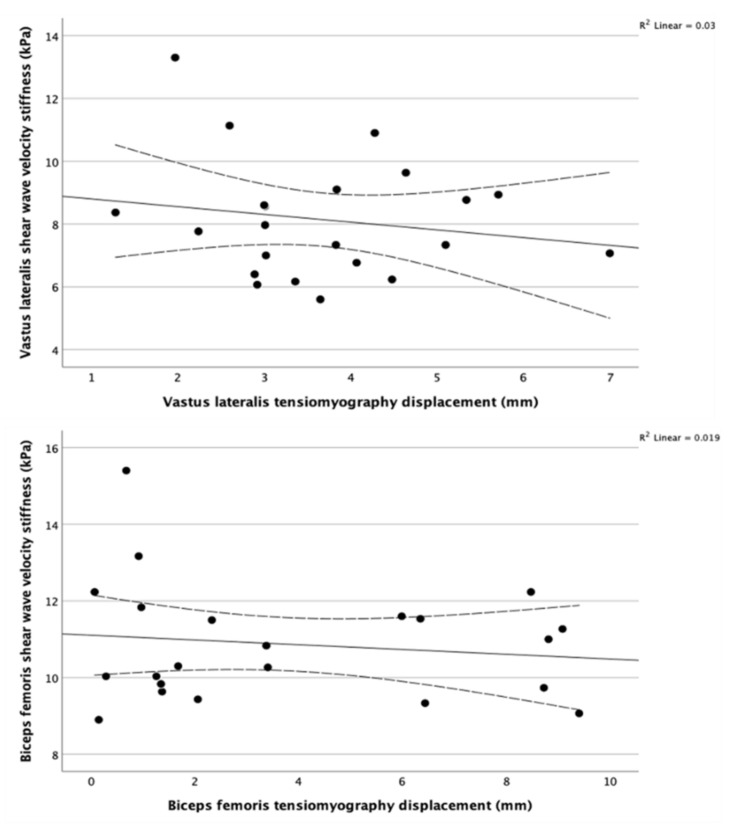
Scatterplots and regression lines for maximum displacement using TMG against shear wave velocity using SWE showing no evident association between them in the vastus lateralis (**top**) and biceps femoris (**bottom**). The dotted lines represent the 95% CI of the mean. The coefficient of determinization in the top right corners confirm the lack of correlation between SWE and TMG for the two muscles.

The correlation coefficients failed to detect any significant correlation (*r* ≤ 0.300, *p* ≥ 0.1) between SWE and TMG variables in the vastus lateralis and biceps femoris. The correlation matrix is listed in Table 2. Figure 3 shows scatterplots between SWE measurements and Dm by TMG. The multiple linear regression for testing the effect size of superficial layers on the TMG measurements was not computed due to the lack of significant correlations (*r* ≤ 0.300, *p* ≥ 0.1) between TMG variables and thicknesses of skin, subcutaneous fat, and fascia. Moreover, the physical activity level did not correlate with any ultrasound or TMG variable (*r* ≤ 0.300, *p* ≥ 0.1). SWE showed excellent intra-operator reproducibility as ICC was 0.964 (95% CI: 0.930, 0.983) for VL and 0.860 (95% CI: 0.729, 0.934) for BF.

## 4. Discussion

The present study aimed to primarily assess the concurrent validity of TMG compared with SWE. No previous studies, to our knowledge, have compared the TMG and SWE measurements in skeletal muscles. It has been proposed that both techniques can infer the same construct, muscle stiffness. Specifically, the displacement (Dm) in TMG has been assumed to be an indirect measure of muscle stiffness [14,23,24,25,26]. Contrary to this assumption, our results observed no association between SWE and TMG. SWE measurements were validated preclinically compared with traditional materials testing techniques [27] and provide accurate responses relative to passive [28] and active forces [29]. SWE was able to detect signs of myositis compared with MRI [30] and identified age-related muscle stiffness changes [6]. Our results do not necessarily invalidate the TMG measurements. Instead, they suggest that SWE and TMG appear to measure different constructs of muscle stiffness properties. More importantly, they should not be considered synonymous when evaluated across different research studies.

A previous study investigated low back pain myofascial trigger points using TMG and strain elastography, an older generation of elastography, which produces qualitative outcomes [31]. They specified multiple active and latent myofascial trigger points as well as additional trigger points before assessing each one using the two techniques. TMG parameters did not yield any statistically significant difference (*p* > 0.05) between active and latent myofascial trigger points and control points. In contrast, elastography was able to detect statistically significant differences (*p* < 0.05) between all point types. This previous study confirms our findings regarding the different constructs each technique is measuring. Other scientists suggest that Dm in TMG relates to the muscle tone in response to the deformations induced by the TMG’s electrical stimulus [32]. Whilst elastography assesses the intrinsic stiffness of the muscle belly relative to the speed of the traveling shear waves.

The results showed that TMG measurements appear to be independent of the superficial tissues, including skin, subcutaneous fat, and fascia. This is also true for SWE. In contrast, Calvo-Lobo et al. found statistically significant but weak correlations of few TMG variables [33]. They studied the TMG variables on the erector spinae muscle and detected weak correlations between skin thickness with Dm (r = −0.329; *p* = 0.020) as well as subcutaneous fat and Tr (r_s_ = −0.369; *p* = 0.008). They also detected a moderate correlation between subcutaneous fat and Dm (r_s_ = −0.668; *p* < 0.001). Similar to our findings, they found no associations between the rest of the variables. These results may indicate a potential difference in superficial tissue responses between the lumbopelvic region and thigh muscles.

Several limitations should be considered when interpreting the results of this study. The sample size was selected based on convenience sampling, which may not be representative of the general population. There was high dropout rate resulting in a small sample size. This also resulted in a sample with a small ratio of female participants. Previous studies, however, indicate no intrinsic differences in muscle stiffness based on sex [34,35].

## 5. Conclusions

Overall, the results showed no significant correlation between SWE and all TMG parameters failing to ascertain the concurrent validity of TMG. This indicates that SWE and TMG appear to measure different mechanical constructs of skeletal muscles. The superficial layers of skin, subcutaneous fat, and muscle fascia had no significant effect on the SWE and TMG measurements. More research is needed to understand the specific muscle stiffness constructs of each measurement parameter in musculoskeletal dysfunctions. Future studies should investigate SWE and TMG on pathological cases to understand the most suitable stiffness construct for detecting different diseases.

## Figures and Tables

**Table 1:** Mean and 95% confidence interval for the ultrasound and tensiomyography variables for vastus lateralis and biceps femoris.

	Vastus Lateralis			Biceps Femoris			*p*-Value
	Mean	SD	95% CI	Mean	SD	95% CI	
Muscle stiffness (kPa)	8.1	1.8	7.3, 8.9	10.8	1.5	10.2, 11.5	**<0.001**
Time of contraction (T_c_) (ms)	26.6	7.3	23.3, 29.8	35.9	18.0	28.9, 43.9	0.050
Sustain time (T_s_) (ms) *	53.7	52.8	50.7, 81.6	159.1	92.3	128.4, 202.2	**<0.001**
Relaxation time (T_r_) (ms) *	20.0	28.9	16.1, 37.9	52.0	65.1	38.9, 89.9	**0.007**
Delay time (T_d_) (ms)	23.1	2.1	22.1, 24.0	25.1	5.8	22.6, 27.7	0.17
Maximal displacement (D_m_) (mm)	3.7	1.3	2.1, 4.2	3.7	3.4	2.2, 5.2	0.90
Muscle thickness (mm)	22.2	3.6	20.6, 23.8	29.5	5.1	27.2, 31.8	<0.001
Skin thickness (mm)	1.2	0.3	1.1, 1.4	1.4	0.4	1.2, 1.6	0.006
Subcutaneous fat thickness (mm)	6.6	3.2	5.2, 8.1	7.2	2.6	6.1, 8.4	0.129
Fascia thickness (mm)	1.2	0.4	1.0, 1.4	1.1	0.3	0.9, 1.2	0.16
Total superficial layer thickness (mm)	9.2	3.2	7.7, 10.6	9.8	2.9	8.5, 11.1	0.11

* Data presented as median with interquartile range. The 95% CI is generated based on 1000 bootstrap samples. The difference was tested using Wilcoxon signed-rank test.

**Table 2:** Correlation coefficients for the association between elastography and the other study variables including tensiomyography variables and superficial layers’ thicknesses.

	Vastus Lateralis			Biceps Femoris		
	Coefficient	*p*-Value	95% CI	Coefficient	*p*-Value	95% CI
Time of contraction (T_c_) (ms)	0.213	0.341	0.213, 0.341	−0.228	0.307	−0.228, 0.307
Sustain time (T_s_) (ms) *	0.043	0.848	0.043, 0.848	0.001	0.996	0.001, 0.996
Relaxation time (T_r_) (ms) *	−0.033	0.883	−0.033, 0.883	−0.187	0.405	−0.187, 0.405
Delay time (T_d_) (ms)	0.145	0.521	0.145, 0.521	−0.129	0.566	−0.129, 0.566
Maximal displacement (D_m_) (mm)	−0.032	0.887	−0.032, 0.887	−0.165	0.463	−0.165, 0.463
Muscle thickness (mm)	0.092	0.661	0.092, 0.661	−0.055	0.792	−0.055, 0.792
Skin thickness (mm)	0.035	0.867	0.035, 0.867	−0.036	0.863	−0.036, 0.863
Subcutaneous fat thickness (mm)	−0.023	0.911	−0.023, 0.911	0.327	0.11	0.327, 0.11
Fascia thickness (mm)	0.193	0.355	0.193, 0.355	0.049	0.817	0.049, 0.817
Total superficial layer thickness (mm)	0.026	0.903	0.026, 0.903	0.298	0.148	0.298, 0.148

* Spearman’s coefficients were used to test the correlations.

## Data Availability

Data are available upon reasonable request submitted to the corresponding author.
